# Nicotine dependence and insula subregions: functional connectivity and cue-induced activation

**DOI:** 10.1038/s41386-023-01528-0

**Published:** 2023-03-03

**Authors:** Dara G. Ghahremani, Jean-Baptiste F. Pochon, Maylen Perez Diaz, Rachel F. Tyndale, Andy C. Dean, Edythe D. London

**Affiliations:** 1 Department of Psychiatry and Biobehavioral Sciences, Semel Institute for Neuroscience and Human Behavior, University of California, Los Angeles, CA USA; 2 Department of Pharmacology & Toxicology and Department of Psychiatry, University of Toronto, 1 King’s College Circle, Toronto, ON Canada; 3 Campbell Family Mental Health Research Institute, Centre for Addiction and Mental Health, Toronto, ON Canada; 4 Brain Research Institute, University of California, Los Angeles, CA USA; 5 Department of Molecular and Medical Pharmacology, University of California, Los Angeles, CA USA

**Keywords:** Addiction, Addiction

## Abstract

Nicotine dependence is a major predictor of relapse in people with Tobacco Use Disorder (TUD). Accordingly, therapies that reduce nicotine dependence may promote sustained abstinence from smoking. The insular cortex has been identified as a promising target in brain-based therapies for TUD, and has three major sub-regions (ventral anterior, dorsal anterior, and posterior) that serve distinct functional networks. How these subregions and associated networks contribute to nicotine dependence is not well understood, and therefore was the focus of this study. Sixty individuals (28 women; 18–45 years old), who smoked cigarettes daily, rated their level of nicotine dependence (on the Fagerström Test for Nicotine Dependence) and, after abstaining from smoking overnight (~12 h), underwent functional magnetic resonance imaging (fMRI) in a resting state. A subset of these participants (*N* = 48) also completing a cue-induced craving task during fMRI. Correlations between nicotine dependence and resting-state functional connectivity (RSFC) and cue-induced activation of the major insular sub-regions were evaluated. Nicotine dependence was negatively correlated with connectivity of the left and right dorsal, and left ventral anterior insula with regions within the superior parietal lobule (SPL), including the left precuneus. No relationship between posterior insula connectivity and nicotine dependence was found. Cue-induced activation in the left dorsal anterior insula was positively associated with nicotine dependence and negatively associated with RSFC of the same region with SPL, suggesting that craving-related responsivity in this subregion was greater among participants who were more dependent. These results may inform therapeutic approaches, such as brain stimulation, which may elicit differential clinical outcomes (e.g., dependence, craving) depending on the insular subnetwork that is targeted.

## Introduction

The use of combustible tobacco products persists as a substantial public health problem, causing >7 million (or 1 in 10) deaths worldwide annually [1]. Among people who try to quit smoking independently, only 3–6% successfully stop for 6–12 months, and most fail within 8 days [2]. Behavioral and pharmacological treatments for Tobacco Use Disorder (TUD) yield limited success [3–5], highlighting a need for novel therapeutic strategies.

Nicotine dependence is a major predictor of relapse in people with TUD. Nicotine dependence measured using the Fagerström Test for Nicotine Dependence (FTND) [6] predicted relapse at 1- and 2-year follow up assessments [7]. In participants maintaining smoking abstinence for a month followed by behavioral and pharmacological therapy, relapse rates at 1-year follow up were higher among those with greater nicotine dependence [8] (measured with FTND). FTND also predicted the success of abstinence imposed by hospitalizations for cardiac problems [9, 10] or pregnancy [11]. These findings suggest that nicotine dependence may be a tangible therapeutic metric and target for determining success in reduction of cigarette smoking or abstinence. Accordingly, elucidating the neural mechanisms of nicotine dependence has the potential to advance TUD treatment [12], particularly with neuroanatomically-based approaches, such as targeted brain stimulation [13, 14]. Further knowledge of the neural systems underlying nicotine dependence is critical for guiding these treatments.

The insula has been identified as a promising therapeutic target for TUD, partly due to clinical evidence for its role in smoking behavior [15, 16]. Patients with insula lesions resulting from strokes markedly reduced their smoking [17–19], and survivors of strokes with damage to the right insula maintained abstinence from smoking for >1 year after hospital discharge [18, 20].

Neuroimaging studies have also indicated the importance of the insula in maintenance of cigarette smoking. Cortical thickness of insular sub-regions is negatively related to nicotine dependence [21–23] and cigarette craving [24]. Resting-state functional connectivity (RSFC) has been used to assess neural systems involved in nicotine dependence [25, 26]. A negative relationship was found between nicotine dependence and RSFC of the anterior cingulate cortex (ACC) with the striatum [27, 28]. Other RSFC studies showed an inverse relationship between nicotine dependence and insula-ACC connectivity [29–31]. Cue-induced craving paradigms have also been used to examine neural systems associated with nicotine dependence [32, 33]—one fMRI study showed a positive relationship between nicotine dependence and activation in the anterior and posterior insula in response to smoking-related cues. The particular insular subregions showing this relationship depended on the condition (e.g., baseline, food cues) used to contrast with the cues condition [32].

The insula has been subdivided into three major sub-regions that serve distinct functions: dorsal anterior, ventral anterior, and posterior [34, 35]. Whereas the posterior portion of the insula connects with sensorimotor integration areas (e.g., pre-motor, supplementary motor cortex), the anterior portion is functionally linked with limbic regions and is a key component of the “salience network”, which includes the ACC [36]. The anterior portion generally serves cognitive and affective functions [37, 38]. Further functional distinctions have been made between dorsal and ventral anterior insula connectivity along cognitive and affective domains, respectively [39, 40]. In addition, dorsal/ventral distinctions have been conceptualized as components of externally- and internally oriented networks—the frontoparietal attention network and the default mode network, respectively [41, 42]. These functional distinctions can be translated to different aspects of TUD. The posterior insula is considered a hub for primary convergence of interoceptive signals [43, 44]; thus, the sensorimotor integration functions of this subregion may be linked to interoceptive aspects of nicotine withdrawal symptoms and craving, especially somatosensory features of the latter. The dorsal/ventral anterior insula distinction along externally/internally oriented networks has been linked to cue-induced vs. spontaneous craving, respectively [42], providing evidence for subregional specificity for different dimensions of craving in TUD. Moreover, the cognitive/affective distinction of dorsal/ventral anterior insula suggests that these regions may differentiate cognitive aspects of the disorder (e.g., planning for the next cigarette) and emotional/affective aspects (e.g., negative affect associated with brief abstinence/withdrawal).

Given the functional heterogeneity of insular subregions, understanding the relationship of each subregion to nicotine dependence can advance efforts to refine neurotherapeutic targets for brain stimulation. The most common non-invasive brain stimulation technique used for TUD is transcranial magnetic stimulation (TMS), but studies of TMS on smoking behavior show weak or no effects [13, 45]. Such discrepancy may, in part, reflect the lack of spatial precision in the brain area stimulated by TMS, which produces stimulation in areas that span across insular subregions and into the prefrontal cortex. Other, more novel non-invasive brain stimulation techniques, such as low intensity focused ultrasound pulsation (LIFUP) [46], can target smaller brain structures and allow for more precise stimulation of insular subregions. However, a better understanding of the differences in these subregions with respect to nicotine dependence is needed to determine optimal insular stimulation targets.

Prior studies of RSFC have attempted to distinguish between connectivity of insular sub-regions with respect to smoking-related behavioral variables [30, 42, 47–49], but only two of them examined RSFC of insula subregions with respect to nicotine dependence [29, 30]. These studies showed negative relationships between connectivity of the dorsal ACC with anterior [29] and posterior [30] insula. Despite this initial evidence for distinctions between subregions, a comprehensive analysis of their contributions is lacking, leaving open the possibility that RSFC of different insular subregions are differentially related to nicotine dependence. We therefore undertook a comprehensive analysis of correlations with connectivity patterns of these insular subregions in a whole-brain analysis. We also tested the relationship between nicotine dependence and activation of insula subregions during cue-induced craving for cigarettes and examined the relationship between this activation and insula RSFC.

In a group of 60 participants who smoked cigarettes daily and maintained overnight abstinence from smoking, we examined the relationship of nicotine dependence with RSFC and cue-induced activation (using task-based fMRI) of three major insular sub-regions (ventral anterior, dorsal anterior, and posterior). Considering the literature, we hypothesized that nicotine dependence would be negatively correlated with connectivity between anterior insula subregions and the ACC (i.e., participants with greater connectivity would show less dependence), and also with connectivity between the posterior insula and sensory-motor integration regions (e.g., supplementary motor area). We also hypothesized that dependence would be positively related to cue-induced activation in subregions of the insula, and that this activation would be related to insula RSFC patterns that are associated with dependence.

## Materials and methods

### Overview of experimental design

Functional magnetic resonance imaging (fMRI) data were collected during the resting state from adults who smoked cigarettes daily and maintained overnight abstinence before testing, which began between 8 and 9 a.m. (all scanning occurred prior to 11 a.m). The study was part of a larger investigation on the brain correlates of smoking behavior and took place between September 2017 and March 2022. Data from a subsample of the participants were used in prior assessments [24, 49]. All participants in the prior resting state fMRI study as well as newly recruited ones were included in this study, which took place at the Semel Institute for Neuroscience and Human Behavior at the University of California, Los Angeles (UCLA). All study procedures were approved by the UCLA Institutional Review Board.

### Participants

Two-hundred seventeen participants were recruited via online and print advertisements. They attended an intake session where they received a detailed explanation of the study procedures, provided written informed consent, and were screened for eligibility. Seventy met all study criteria and completed all procedures. Inclusion criteria were as follows: age of 18–45 years, generally good health, self-report of smoking at least 4 cigarettes per day for at least 1 year. Recent smoking history was verified during the intake session using a urine cotinine test (ACCUTEST Urine Cotinine Test, Jant Pharmacal Corp., Encino, CA, score ≥3, cotinine ≥200 ng/ml). Exclusion criteria were positive urine tests for drugs of abuse other than nicotine or tetrahydrocannabinol, self-report of consuming ≥10 alcoholic drinks per week, any current psychiatric disorder other than TUD assessed via the Mini International Neuropsychiatric Interview for DSM-5 [50, 51], history of neurological injury, and using electronic cigarettes, cigars, snuff, or chewing tobacco >3 times a month.

### Verification of drug, alcohol abstinence and nicotine dependence

On the testing day, overnight (~12 h) abstinence from smoking was assessed with the Micro+ Smokerlyzer® breath carbon monoxide (CO) monitor (Bedford Scientific Ltd., Maidstone, Kent, UK). Participants were considered to have abstained on the day of testing if they either had a CO level of <10 ppm or showed a 25% reduction from the CO measurement at the intake visit. Abstinence from cocaine, opiates, benzodiazepines, and amphetamines was verified with a five-panel urine drug test (Drugs of Abuse Test Insta-view®, Alfa Scientific Designs Inc., Poway, CA). Alcohol abstinence was verified using a breathalyzer (Alco-Sensor FST®, Intoximeters, Inc., St. Louis, MO). Recent abstinence from cannabis use was verified with the Dräger DrugTest® 5000 saliva test (Dräger, Inc., Houston, TX). Nicotine dependence was measured during the intake session using the FTND [52].

### MRI data acquisition

All images were acquired on a 3-Tesla PRISMA (Siemens) MRI scanner using a 32-channel head coil receiver. The resting state imaging protocol consisted of the continuous acquisition of 738 Echo-planar Image (EPI) volumes over a period of 9 min and 50 s. A multiband accelerated EPI pulse sequence (factor 8) was used, allowing us to acquire 72 axial slices during a repetition time (TR) of 800 ms with a 104 × 104 matrix. Resolution was 2 × 2 × 2 mm^3^, echo time (TE) was 37 ms, and the flip angle was 52 degrees. Participants were asked to keep their eyes open and to look at a black screen during the resting state scan. For the fMRI task, a multi-echo EPI (ME-EPI) sequence was used (TE1 = 13.80 ms, TE2 = 35.94 ms, TE3 = 58.08 ms) with a multiband acceleration factor of four and Generalized Auto-calibrating Partially Parallel Acquisition acceleration factor of 2 (TR = 1.462 s, flip angle = 67 degrees, 280 volumes). Each volume consisted of 64 interleaved axial slices (2 mm cubic, 104 × 96 matrix). The multiecho sequence was used to increase the signal-to-noise ratio by reducing effects of non-TE dependent nuisance fluctuations such as motion and hardware instabilities [53] and by combining optimally the signals from the different echoes [54]. The structural T1-weighted images were obtained using a magnetization prepared rapid gradient echo sequence with the following parameters: isovoxel 0.8 mm^3^, FOV = 240 × 256 mm^2^, TE = 2.24 ms, TR = 2400 ms; flip angle = 8°; 208 sagittal slices.

### Cue-induced craving task

The task paradigm consisted of the presentation of short video clips, created by a professional film maker, that included both indoor and outdoor scenes in which young adults engaged in identical activities either while smoking (smoking cues) or not (neutral cues). These videos were used in two prior studies [55, 56]. Individuals in the videos were professional actors who provided consent for publication of their images. Six smoking cues and six neutral cues videos were presented. Each video clip was ~15 s in length (mean 14.97 s ± 0.27) and was followed by a 1-s blank screen, after which participants were shown the following sentence: “What is your urge to smoke right now?”. Participants had 10 s to indicate their response by using a trackball (Trackball2, Current Designs, Inc., Philadelphia, PA) to position the cursor on a radially arranged visual scale consisting of seven digits: “1” = “No urge”, “7” = “Extreme urge” (see Supplementary Fig. 2 for schematic and further task details). After an 8-s inter-stimulus interval (ISI), the next video sequence was presented. Each trial, including the ISI, was ~34 s in duration. Video presentation order was pseudorandom across participants, such that number of consecutive videos of the same type (smoking, neutral) did not exceed three. The presentation and timing of all stimuli and collection of responses were programmed using Matlab (Mathworks, Natick, MA) and the Psychtoolbox (3.0.14, Rev. 8424) (www.psychtoolbox.org) on an Apple MacBook Pro laptop (MacOS 10.12.6, Apple Computers, Cupertino, CA). During scanning, visual stimuli were presented using a projector at the rear of the bore of the scanner, with participants viewing them via a mirror mounted on the head coil.

### MRI data pre-processing

Image preprocessing for resting state data were mostly conducted with FSL (5.0.9). The initial stages included rigid body realignment to correct for head movements within each scanning run, skull removal, and non-linear registration to the Montreal Neurological Institute (MNI) template. Initial motion cleaning and noise reduction were performed using a 24-parameter linear regression model that included six motion parameters (3 translational dimensions along *X*, *Y* and *Z* axes and 3 rotational dimensions: “pitch”, “roll” and “yaw”), the temporal derivatives of these parameters, and the quadratic of all parameters [57]. Mean frame displacement (FD) and the variance of signal change from the average signal (DVARS) of the raw images were estimated. A null sampling distribution of DVARS was used to identify frames with excessive variance at *p* < 0.05 [58]; frames with FD > 0.45 mm were also flagged. These frames as well as the one located in time just prior (*t* − 1) and two just after (*t* + 1 and *t* + 2) were included in a censoring temporal mask for data interpolation: a least-squares spectral decomposition of the uncensored data was performed to reconstitute data of the censored timepoints [see methods in [59]]. The uncensored data defined the frequency characteristics of signals that then replaced the censored data. This step aimed at minimizing the contamination of the signal from the censored frames during frequency filtering. The interpolated signal was then demeaned, detrended, and filtered using a bandpass filter (0.009–0.08 Hz), after which the interpolated timepoints were censored. Participants with >50% frames censored (i.e., those with <5 min of remaining resting state data) were excluded from analyses. To reduce the contribution from non-neuronal noise, the minimal number of principle components that explained at least 50% of the variance of mean signal extracted from white matter and cerebrospinal fluid were evaluated and regressed out from the signal [aCompCor50, [60]]. Volumes were then spatially smoothed with a Gaussian filter using a 5-mm FWHM kernel. Each voxel was normalized to a mean value of 100 (SD = 1) to transform the data to Pearson’s correlation coefficients (*r*).

The task-based fMRI data preprocessing differed slightly from that used for resting state data because of the ME-EPI sequence used. Data were first organized in the Brain Imaging Data Structure format [61] and processed using the fMRIPrep pipeline (version 20.2.5) for ME-EPI data [62]. Preprocessing was as follows: a reference volume and its skull-stripped version were generated from the shortest echo of the BOLD run using a custom method in fMRIPrep. The BOLD reference was then co-registered to the T1w reference using “bbregister” (FreeSurfer), which implements boundary-based registration. Co-registration was configured with six degrees of freedom. Head-motion parameters with respect to the BOLD reference (transformation matrices, and six corresponding rotation and translation parameters) were estimated before spatiotemporal filtering using FSL’s MCFLIRT. BOLD runs were slice-time corrected using “3dTshift” (AFNI 20170207). The BOLD time-series (including slice-timing correction when applied) were resampled onto their original, native space by applying the transforms to correct for head-motion. A T2* map was estimated from the preprocessed BOLD images by fitting to a mono-exponential signal decay model with nonlinear regression, using T2*/S0 estimates from a log-linear regression fit as initial values. For each voxel, the maximal number of echoes with reliable signal in that voxel were used to fit the model. The calculated T2* map was then used to optimally combine preprocessed BOLD across echoes following the method described in [54]. The preprocessed BOLD time-series were then resampled into standard space (MNI152NLin2009cAsym) using the nonlinear registration calculated between the T1w image and a template (ANTs 2.1.0). The confounding time-series FD and DVARS were calculated based on the preprocessed BOLD images. Images with >0.5 mm FD or >1.5 standardized DVARS were annotated as motion outliers. All analyses were performed on Linux (CentOS release 6.10) using FSL (5.0.9), MATLAB (8.6), R (3.6.0) and FreeSurfer (6.0.0).

### Resting-state fMRI seed-based analysis

To minimize bias, we used a statistically conservative voxel-wise whole-brain analytic approach rather than restricting to a priori-selected target regions or networks. On each of the two hemispheres of the brain, three insula seeds (ventral-anterior, dorsal-anterior, and posterior), were defined for RSFC analyses (Supplementary Fig. 1). To define the anterior insula, we compared anatomical landmarks from a probabilistic atlas [63] to RSFC-based parcellations of the insula [34, 35]. From these studies, we defined the ventral anterior insula parcel as the anterior inferior insular cortex (which includes the apex, the limen, and the transverse gyrus). The dorsal anterior insula was defined as the anterior and middle short gyri. The precentral sulcus was used to segment the anterior from the posterior insula. Using these landmarks, we manually determined the anterior insular subdivisions (dorsal and ventral, left and right) from the MNI152 template.

To evaluate RSFC between the insula seeds and other brain regions, the time series from each seed was extracted, and its first normalized eigen vector (mean = 100, SD = 1; to facilitate computation of Pearson’s *r*) was used as a regressor in an ordinary least squares linear regression analysis on every voxel (as implemented in “film_gls” in FEAT). The parameter estimates of the model, corresponding to the Pearson’s correlation coefficient (since data were previously normalized), were z-transformed to improve data normality.

The resulting z-transformed images were used in multi-level mixed effects models for group analyses with FMRIB’s Local Analysis of Mixed Effects (FLAME1) with outlier deweighting, testing for the effect of nicotine dependence on RSFC for each seed. Specifically, two separate models were tested. The model included the total score of the FTND as the independent variable of interest.

To account for differences in motion during scanning between participants, the mean FD value was included as a covariate in all models, in addition to age. Results were cluster-corrected for multiple comparisons using a voxel-height threshold of *p* < 0.001 (*Z* > 3.1) and cluster threshold of *p* < 0.05 as recommended per Eklund et al. [64]. The coordinates reported here correspond to the peak voxel within a given cluster in MNI coordinate space.

### Cue-induced craving task fMRI analysis

Individual preprocessed BOLD time-series were analyzed using the General Linear Model within FSL’s FEAT (FSL 5.0.9). The design matrix used included four explanatory variables: (1) smoking and (2) non-smoking cues (i.e., videos), and the rating phase after (3) smoking and (4) non-smoking cues. Delta functions for each variable were defined as (1) the start and end of each video presentation and (2) the onset of the rating prompt followed by the time in which the response was made. These boxcar shaped delta functions were then convolved with a double gamma HRF function and used as the explanatory variables. The 24 motion parameters estimated during preprocessing were included in the model, as were all frames flagged as motion outliers. Low frequency fluctuation of the signal was removed using a 200-s high-pass filter. The contrast images of the model were spatially smoothed with a 5 mm FWHM kernel. The contrast of smoking cues minus neutral cues was used for the group analysis. To parallel the RS seed analysis, we performed region of interest analyses of contrast estimates. The same a priori-defined insular subregions used as seeds for the resting state analyses (Supplementary Fig. 1) were assessed using the General Linear Model in Jamovi [65] with age and mean frame displacement (mFD) as covariates. We also performed a voxel wise whole brain group-level mixed-effects analysis (FLAME1 with outlier deweighting). The FTND score for each participant was used in this model, as well as age and mFD. Statistical maps reflecting (1) the mean effect of smoking cue and (2) the influence of nicotine dependence (FTND) on this effect were cluster-corrected for multiple comparisons (voxel height threshold: *Z* > 3.1 (*p* < 0.001), cluster significance *p* < 0.05). All analyses at the group level were performed and reported in MNI space.

## Results

### Participant characteristics

Seventy two adults who endorsed daily cigarette smoking (and met study inclusion criteria) underwent resting state fMRI scanning. Among this group, twelve subjects were excluded for not meeting abstinence criteria (*N* = 7) or had excessive motion during the rest scan (*N* = 5). The final sample of participants with resting state data included 60 individuals whose characteristics (sex, age, level of nicotine dependence, cigarette use, and exposure) are shown in Table 1. The same group of 60 participants received the cue-induced craving task; however, nine participants were excluded due to technical problems during scanning (i.e., behavioral response equipment failures and visual display issues), and three participants were excluded for excessive motion (>0.5 mm FD). In all, data from 48 participants were used in the final analysis of task fMRI data—all of whom also received resting state scans. Table 1 shows participant characteristics for participants whose cue-induced craving task data were used; characteristics did not significantly differ from the resting state sample (*p*s > 0.5).

**Table 1:** Participant characteristics for subgroups with resting state and cue-induced craving fMRI data.

Characteristic	Resting state fMRI		Cue-induced craving fMRI	
*N*	60		48	
Sex	32 M/28 F		24 M/24 F	
Ethnicity				
Not Hispanic or Latino	80.0%		79.2%	
Hispanic or Latino	15.0%		18.8%	
Unknown	5.0%		2.1%	
Race				
White	53.3%		54.2%	
Black/African	23.3%		25.0%	
Mixed	6.7%		4.2%	
Hawaiian/Pacific Islander	3.3%		4.2%	
Asian	1.7%		2.1%	
Unknown	11.7%		10.4%	
	Mean (range)	SD	Mean (range)	SD
Age (years)	32.8	7.1	32.9	7.3
Nicotine dependence (FTND^a^)	4.1 (0–9)	2.1	4.1 (0–9)	2.2
Tobacco/current use (cigarettes/day)	11.7	5.1	11.4	4.9
Tobacco/lifetime exposure (pack-years)	8.2	6.1	7.8	5.8
Alcohol consumption				
*N* with >0 drinks/week (drinks/week)	(*N* = 14) 5.8	2.5	(*N* = 12) 5.7	2.7
Cannabis consumption				
*N* with >0 gram/week (grams/week)	(*N* = 19) 4.4	4.6	(*N* = 15) 4.3	5.1
Craving ratings during cue presentation^b^				
Smoking cues			4.9	1.8
Neutral cues			4.0	1.7

Groups had overlapping participants and did not differ by age, ethnicity, race, FTND, cigarettes per day, or packs-years, alcohol and cannabis consumption (*t*-test, *p*s > 0.05).

^a^Fagerström Test of Nicotine Dependence.

^b^Craving ratings significantly differed across cue-types (*t*-test, *p*s < 0.05).

### RSFC of insular subregions and cue-induced activation

Examining the mean connectivity of each insular subregion, we found a distinction between anterior and posterior insula connectivity patterns, but very little difference between ventral and dorsal anterior insula connectivity (Supplementary Fig. 3). With respect to the primary anterior insula connectivity target that was related to FTND—the superior parietal lobule (SPL) (see results below)—we observed significant RSFC of all anterior insular seeds with SPL at our default threshold (*p* < 0.001), except for the correlation with the left ventral anterior seed, which was significant at *p* < 0.01.

**Fig. 1:**
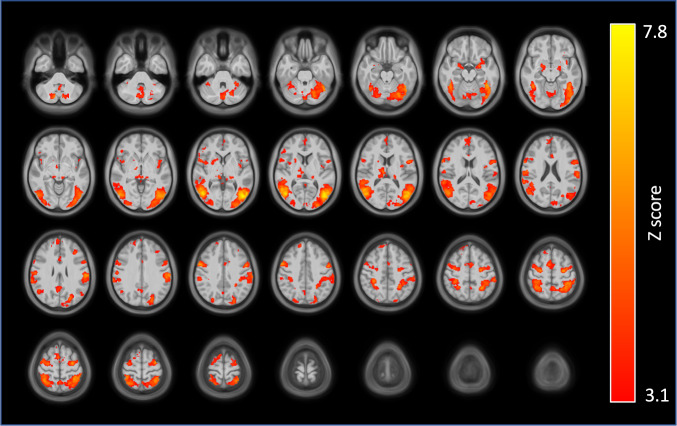
Whole-brain, voxel-wise cluster-corrected results from the contrast of smoking vs. neutral cues. Regions of activation included amygdala, occipito-temporal cortex, medial prefrontal cortex, frontal eye fields, intraparietal sulcus, superior parietal lobule, and insula (mostly posterior). Figure shows thresholded statistical map (*Z* > 3.1 (*p* < 0.001), cluster corrected) displayed on the average of spatially normalized T1-weighted images across participants (*N* = 48). Brain images are presented in radiological convention (right = left).

Whole-brain voxel-wise results from the contrast of smoking vs. neutral cues indicated several regions of activation, including amygdala, occipito-temporal cortex, medial prefrontal cortex, frontal eye fields, intraparietal sulcus, and SPL (mainly within the precuneus), and insula (Fig. 1). Insula clusters were found in mostly anterior but some posterior left and right subregions.

### Nicotine dependence and RSFC of insular subregions

**Fig. 2:**
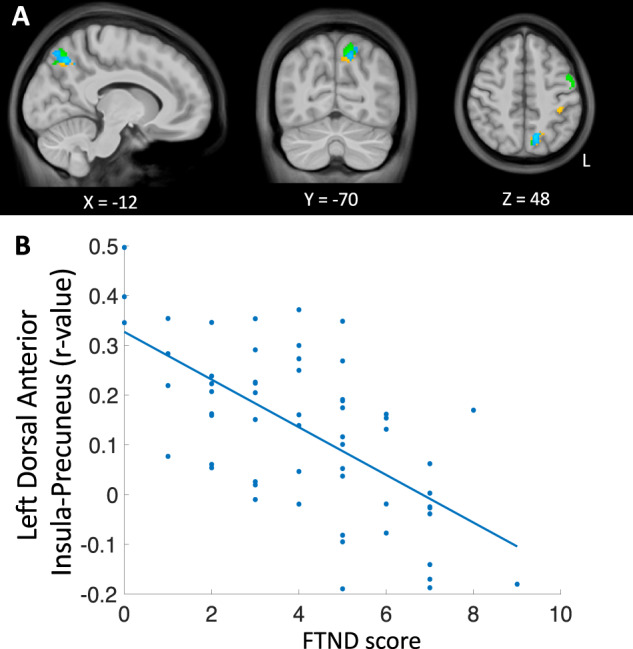
Relationship between nicotine dependence (FTND total score) and anterior insula-superior parietal lobule functional connectivity. FTND total score correlated negatively with connectivity of several insular subregions and clusters within the superior parietal lobule (SPL). Figure shows thresholded statistical maps [voxel height: *Z* > 3.1 (*p* < 0.001), cluster threshold: *p* < 0.05] indicating insula connectivity targets in left precuneus/SPL. Insula seeds are represented by different colors: left (orange) and right (blue) dorsal anterior insula and left (green) ventral anterior insula (the overlapping cluster from the right ventral anterior insula seed is not shown). Results are summarized in Table 2. Brain images are presented in radiological convention (right = left) (“L” in figure indicates left side of the brain). **B** Scatterplot of data extracted from left precuneus cluster (blue in **A**) from the left dorsal anterior insula connectivity maps of individual participants are shown along with linear fit to illustrate the negative direction of the relationship between FTND and functional connectivity. The data points appeared similarly for connectivity other anterior insula seed regions.

Testing the association of the FTND total score with RSFC between the insula seeds and the rest of the brain revealed relationships for the anterior but not posterior insular seeds. The connectivity target regions were mostly in the SPL for all anterior seed regions, and only negative relationships were found (Table 2 and Fig. 2). Specifically, we observed negative relationships between FTND and RSFC of the left and right dorsal and left ventral anterior insula with medial portions of the SPL, including the left precuneus (*x* = −10, *y* = −72, *z* = 50), and more lateral areas of the SPL. The left ventral anterior insula also showed the precuneus target when assessed at an uncorrected threshold (*p* < 0.001). For both the left and right ventral and right dorsal anterior insula seeds, a common lateral SPL target was observed (*x* = 28, *y* = −50, *z* = 70).

**Table 2:** Correlation of nicotine dependence (FTND scores) with resting state functional connectivity and cue-induced activation of insular subregions.

Seed	Connectivity target/ activation cluster^a^	Laterality	Cluster volume (voxels)	*Z* (max)^b^	*X*^c^	*Y*^c^	*Z*^c^
Correlation of FTND with resting state functional connectivity of insular subregions							
Left dorsal anterior insula							
Superior parietal lobule/Precuneus		L	133	4.91	−10	−72	50
Postcentral sulcus		L	80	4.22	−40	−34	46
Right dorsal anterior insula							
Superior parietal lobule		R	236	4.19	22	−50	66
Superior parietal lobule (Precuneus)		L	141	5.34	−10	−72	50
Superior parietal lobule		L	129	4.33	−28	−46	66
Superior parietal lobule (Precuneus)		R	107	4.59	6	−58	62
Precentral gyrus		R	73	4.66	20	−12	68
Left ventral anterior insula							
Precentral gyrus		L	171	4.48	−52	4	44
Superior parietal lobule (Intraparietal sulcus/Precuneus)		L	170	4.61	−14	−68	52
Superior parietal lobule		R	143	4.35	28	−50	70
Right ventral anterior insula							
Superior parietal lobule		R	113	4.35	28	−50	70
Correlation of FTND with cue-induced fMRI activation (smoking cues vs. neutral cues)							
Middle frontal gyrus		L	115	4.5	−28	38	18

Whole-brain voxel-wise resting state functional connectivity targets and smoking cue-induced fMRI activation clusters are presented. All results were cluster corrected (voxel height: *Z* > 3.1 (*p* < 0.001), cluster threshold: *p* < 0.05).

^a^Anatomical labels determined via parcellations from the atlas by Destrieux et al. [87].

^b^*Z*-statistic of peak voxel.

^c^MNI coordinates of peak voxel within cluster.

### Nicotine dependence and cue-induced activation in insular subregions

Region-of-interest analyses of the a priori-defined insular subregions indicated that cue-induced activation in the right and left dorsal anterior insula showed positive correlations with FTND (*p* < 0.05, Table 3). These effects would not survive the Bonferroni correction for the six a priori tests and should therefore be cautiously interpreted. The whole-brain voxel-wise analysis revealed a positive correlation of nicotine dependence with cue-induced activation in the left middle frontal gyrus (Supplementary Fig. 4). Specifically, the interaction of cue-type (smoking/neutral) and FTND showed clusters in this region. Using an uncorrected threshold (*p* < 0.001 uncorrected for cluster size), we observed a cluster (18 contiguous voxels) within the left anterior dorsal insula (*X* = −32, *Y* = 10, *Z* = 4; peak *Z* = 4.16). We observed no negative relationships between FTND and activation from the smoking vs. neutral cues contrast, nor did we observe significant relationships between craving ratings during the task (i.e., mean smoking minus mean neutral cue ratings, mean of all cues combined) and insular activation (Table 1).

**Table 3:** Correlation of nicotine dependence (FTND scores) with cue-induced activation (smoking vs. neutral cues) of insular subregions.

Insula subregion	SS	*F*	*p*	Effect size (*η*²*p*)
L dorsal anterior	3777	6.75	0.012^a^	0.133
R dorsal anterior	1641	5.08	0.029^a^	0.104
L ventral anterior	1301	2.27	0.139	0.049
R ventral anterior	854	2.17	0.148	0.047
L posterior	490	1.21	0.278	0.027
R posterior	538	1.82	0.184	0.040

Statistical values for each subregion are from separate linear models that included age and mean FD as covariates. General linear model details in “Methods”.

*SS* sums of squares.

^a^Statistically significant at *p* < 0.05.

### Post hoc analysis of FTND items with insular RSFC and cue-induced activation

To determine if specific item(s) from the FTND questionnaire drove the relationships between the FTND total score and insular RSFC and cue-induced activation, we conducted several post hoc analyses that included responses to each of the six FTND items in the same model, including age and mean FD as covariates. Separate models were run for each insular subregion and each of the two imaging outcomes (RSFC, activation). For RSFC, three FTND items significantly correlated negatively with insula-SPL RSFC (Supplementary Table 1). The first FTND item (FTND1), assessing the time until the first cigarette of the day after waking, was significantly related to RSFC of all insular subregions with SPL, whereas the fourth item (FTND4), assessing the number of cigarettes smoked per day, was significantly related to bilateral dorsal anterior and left ventral anterior insula RSFC with SPL (Supplementary Fig. 5). The third item (FTND3), asking whether participants hate to give up cigarettes smoked in the morning vs. other times, was only significantly negatively associated with dorsal anterior RSFC with SPL. For cue-induced activation, all insular subregions significantly correlated positively with the 5th item (FTND5), assessing whether or not participants smoke more frequently during the first hours after waking than during the rest of the day. In addition, activation in the left ventral anterior insula showed a significant correlation with FTND4.

### Relationship between insular RSFC and cue-induced activation

**Fig. 3:**
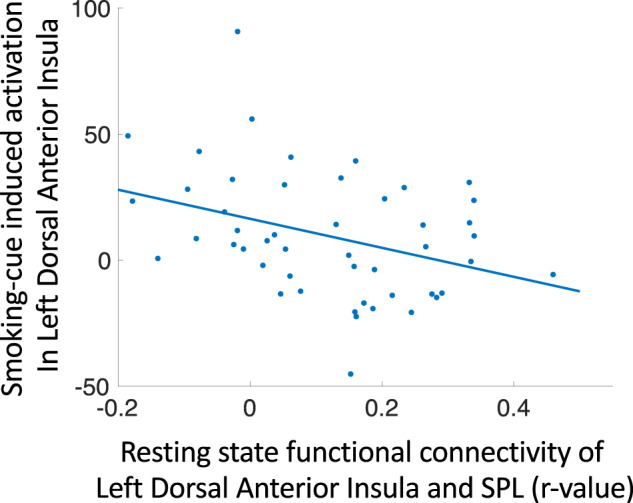
Relationship between left dorsal anterior insula-SPL resting state functional connectivity and cue-induced activation (smoking vs. neutral cues) in the same insular subregion (*p* < 0.013). Further statistics are provided in Supplementary Table 2.

We completed an additional post hoc analysis to determine whether RSFC of anterior insula seeds and the SPL are related to cue-induced activation within the respective anterior insular subregions. Mean RSFC of each seed and its SPL target were used as independent variables in four separate linear models, using the smoking vs. neutral cue contrast estimates (averaged within each seed) as dependent variables (controlling for age and mFD). We found that the activation of the left dorsal anterior insula was negatively correlated with RSFC between the left dorsal anterior insula and the SPL (*p* < 0.013; Fig. 3 and Supplementary Table 2).

### Controlling for cannabis co-use and effects of sex

Since a portion (one-third) of the participants were co-users of cannabis, we conducted post hoc analyses to determine if the RSFC and cue-induced activation results were influenced by cannabis use. We included days of cannabis use in the last 30 days and amount used per week as covariates in a linear model for each insular subregion to determine effects on connectivity and cue-induced activation. We also examined effects of sex given known sex differences in the relationship between nicotine dependence and cigarette craving and in anterior insular thickness [24]. No significant effects were found for any of these covariates (Supplementary Table 3).

## Discussion

This study showed relationships between nicotine dependence and RSFC of subregions within the anterior insula. Specifically, seed-based whole-brain RSFC analyses, using six insular subregions as seeds, indicated a negative association with connectivity of the anterior insula and the SPL. The precuneus, a region along the medial wall of the SPL, and a cluster in the right lateral SPL were the most common connectivity targets among the anterior insula seeds that showed a relationship with dependence. Moreover, we observed a positive relationship between cue-induced activation in the dorsal anterior insula and nicotine dependence, and an inverse relationship was observed between activation in this region and RSFC with SPL. A subset of FTND items primarily drove the FTND relationships for all or some of insular subregional RSFC with SPL and cue-induced activation.

These results suggest potential targets for neuromodulation studies. Specifically, modulation of RSFC in anterior insula-SPL circuits, by stimulating the anterior insula, SPL, or intermediary regions connecting this circuit, may potentially impact nicotine dependence. In this regard, promising results from LIFUP stimulation of subcortical regions indicate modulation of cortical networks, illustrating impact of stimulation of a local region on its related circuitry [66]. The cue-induced activation results also suggest that the dorsal anterior insula may be a potential target for modulating the craving response in those who have relatively high levels of nicotine dependence. Whether modulation of these targets can impact nicotine dependence and craving remains to be empirically determined.

Although we hypothesized a relationship between nicotine dependence and connectivity of anterior insula subregions with limbic regions and components of the salience network (i.e., ACC), we found a relationship with connectivity to SPL regions, brain areas that are not included among limbic or salience network structures but are involved in cognitive and affective function. The precuneus has been associated with self-referential processing, empathy, and episodic memory retrieval [67], and has a central role in the default mode network [68, 69]. It is a primary brain region activated during smoking cue-induced activation, as indicated by meta-analyses [70]. Precuneus activation during presentation of smoking- and alcohol-related cues was positively associated with nicotine and alcohol dependence, respectively [71]. Moreover, in individuals who smoke cigarettes, anterior insula-precuneus connectivity, similar to the connectivity pattern observed in this study, was correlated with cue-induced craving [72]. Both the prior and current findings suggest a role for the interaction of the precuneus with the anterior insula in maintenance of nicotine dependence, especially aspects that involve self-referential processes. The lateral SPL and the precuneus showed similar effects. With its role in visual-spatial attention [73, 74], weakened anterior insula connectivity with lateral SPL in those with higher levels of dependence may reflect disrupted coordination of attentional processing during periods of abstinence.

The regional specificity of the relationship between nicotine dependence and RSFC of anterior vs. posterior insula suggests that nicotine dependence is more associated with affective/cognitive vs. somatosensory aspects of interoception. Given the role of the posterior insula as the “primary interoceptive cortex” [43, 44], receiving input about somatosensory states of the body, our findings suggest that dependence is not significantly associated with these primary interoceptive functions, but mostly with higher order affective/cognitive domains served by the anterior insula [39, 40]. Example cognitive aspects of interoception involved in nicotine dependence may include linking bodily sensations related to craving to planning for nicotine consumption (e.g., determining the next occasion to smoke), and affective aspects may include generation of negative affective states based on bodily sensations related to brief abstinence/withdrawal.

Our results are partially consistent with previous insula RSFC studies of nicotine dependence, which found associations with the ACC. One study of young participants (15–24 years old) who did not abstain from smoking prior to scanning found a negative relationship between anterior insula-ACC RSFC and nicotine dependence [29]. However, ACC was selected as the connectivity target of interest post hoc, based on an analysis that showed greater anterior insula-ACC connectivity in individuals who smoked vs. those who did not. Another study similarly found a negative association between dependence and insula-ACC connectivity [31], but it used the whole, bilateral insula as a seed, eliminating the possibility of differentiating effects based on subregions and laterality. Lastly, a third study found a negative relationship between nicotine dependence and posterior insula-ACC connectivity in individuals with schizophrenia, as well as those without psychiatric diagnoses [30]. The current study may not have found significant results involving the ACC due to differences in analytic methodology. We used whole-brain voxel-wise RSFC analyses with strict multiple comparison correction. Yet, even when lowering the voxel-height threshold to *p* < 0.05 (from *p* < 0.001), we did not find significant clusters within the ACC after applying cluster-based correction. Another possible reason for the absence of a positive ACC finding may be our use of rigorous motion-cleaning approaches [59, 75, 76]—considered essential for removing artifacts that may lead to spurious correlations [77]. The use of a simultaneous multi-slice sequence may have been another point of discrepancy with previous findings, especially given the relatively high multiband factor (MB = 8) used. Although spatial biases in correlations due to noise amplification in seed-based RSFC analyses increases with higher MB factors, application of temporal filtering, as we have done here, decreases these biases [78]. Despite the possibility of reduced effect sizes with temporal filtering, we did not observe results in ACC, even with lower statistical thresholds. Direct comparisons of single- and multi-band acquisition sequences with various factors would be needed to determine the potential effects of MR acquisition parameters on our findings.

We found a relationship between cue-induced activation and dependence in bilateral dorsal anterior insula, considered important for updating motivational states with respect to specific actions [40]. The relationship we observed suggests that the region is more sensitive to switching between appetitive stimuli (i.e., smoking cues) and neutral stimuli in those who are more dependent on nicotine.

Our cue-induced activation findings disagree slightly with those of prior studies. One study found a relationship of nicotine dependence with cue-induced activation in the right posterior insula when comparing cues vs. baseline (similar contrast used in our study), and in left anterior and posterior insula in a comparison of responses to smoking vs. food cues [32]. In addition, we did not find a relationship between nicotine dependence and cue-induced activation in the precuneus as previously observed [71]. These discrepancies may be due to a number of factors related to differences in neuroimaging methods and analytic practices. These include stricter statistical thresholding procedures adopted by the neuroimaging community since the publication of the earlier studies [64] as well as greater noise reduction offered by multi-echo BOLD imaging acquisition and preprocessing approaches used here [54].

Analysis of the FTND items revealed that some contributed significantly to relationships with RSFC. Time until the first cigarette of the day and number of cigarettes per day were associated with RSFC of most anterior insula subregions with SPL (right ventral anterior insula RSFC was not related to cigarettes per day). These two FTND items comprise the Heaviness of Smoking Index, another measure that predicts success of smoking cessation [79, 80]. Anterior insula-SPL RSFC may contribute to aspects of nicotine dependence that influence success of smoking cessation. We also observed a strong relationship between cue-induced anterior insula (all subregions) and frequency of smoking during the first hours after waking. Although the implications of this finding are not entirely clear, cognitive/affective aspects of interoception served by the anterior insula may be related to a sense of urgency to smoke.

Observation of a negative relationship between left dorsal anterior insula-SPL RSFC and cue-induced activation in the same subregion is in line with the assumption that those with greater nicotine dependence (lower anterior insula-SPL RSFC) would respond more strongly to smoking cues (i.e., have greater craving) during brief abstinence than those with less dependence. This link between “spontaneous” anterior insula-SPL RSFC and cue-induced activation suggests that dorsal anterior insula circuitry may be an important target for addressing both nicotine dependence and craving.

Brain stimulation is currently viewed as a promising therapy for smoking cessation [81, 82] and studies targeting the insula have demonstrated mixed success in affecting smoking-related variables, such as craving and withdrawal [13, 16]. TMS has received clearance by the U.S. Food and Drug Administration for smoking cessation [83], and while several studies have shown encouraging results, including one indicating that high-frequency deep TMS to the insula and prefrontal cortex reduces nicotine dependence [84], others have shown weak or no effects [13, 45]. By highlighting the relevance of insular subregional RSFC patterns, which are weaker in strength in those who have greater nicotine dependence, this work provides additional relevant targets for future stimulation studies. For example, stimulation studies may not only choose to target the anterior insula, but also regions within the SPL with the aim of increasing RSFC within the relevant circuitry we’ve identified. Although it is not known whether such anatomical specificity is important for successful therapeutic outcomes, our results suggest this possibility. Stimulation techniques that provide greater spatial resolution (e.g., LIFUP) than TMS would be required to test this specificity. We did not find effects of co-use of cannabis and sex, suggesting that therapeutic interventions would be effective for those who use other substances in addition to nicotine and robust across the sexes.

This study is limited in that it cannot determine whether weak insular connectivity is a cause or consequence of nicotine dependence although pre-clinical studies have suggested a potential causal role of insular connectivity on dependence. In a data-driven study of rats, an insular-frontal network was predictive of later nicotine dependence [85]. It remains to be determined if a similar finding will emerge from longitudinal human studies, such as the Adolescent Brain Cognitive Development study [86].

Overall, we provide evidence for the association of nicotine dependence with weak RSFC and greater cue-induced activation of distinct insula subregions, mainly, dorsal anterior insula-right SPL connectivity and dorsal anterior insula activation, respectively. This regional specificity within the insula highlights the heterogeneity of the insula with respect to neural processes involved in maintenance of smoking and suggests multiple potential targets for brain-based therapies that address nicotine addiction.

## Supplementary information

Supplemenatary Materials

## Data Availability

All self-report, toxicology, and summary fMRI data discussed in this manuscript, as well as the code used for statistical analyses, are publicly available from the Open Science Framework web site under project title, “Nicotine dependence and functional connectivity and activation of insular cortex subregions” (https://osf.io/24tkf/).
