# Supplementary material for: Nicotine dependence and insula subregions: functional connectivity and cue-induced activation
Source: Neuropsychopharmacology. 2023 Mar 3;48(6):936–45. doi: 10.1038/s41386-023-01528-0 (PMC10156746; doi:10.1038/s41386-023-01528-0)
Supplement: Supplementary file 1 — Supplemenatary Materials [file 41386_2023_1528_MOESM1_ESM.pdf]

SUPPLEMENTARY FIGURES & TABLES

Ghahremani, Pochon, et al. *Nicotine dependence and insula subregions: functional connectivity and cue-induced activation*.

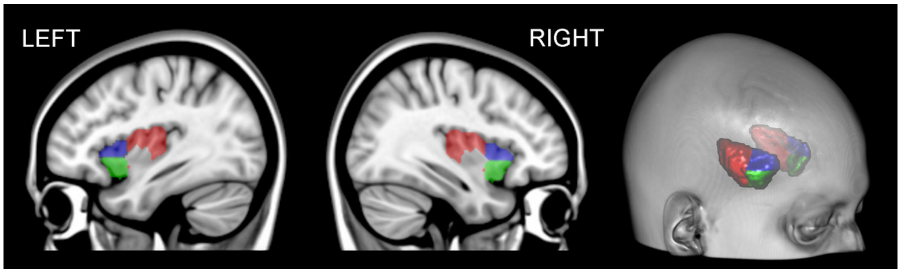

Supplementary Figure 1. Insula sub-regions used as seeds for resting state functional connectivity analyses. Red – ventral anterior insula, Blue – dorsal anterior insula, Green – posterior Insula.

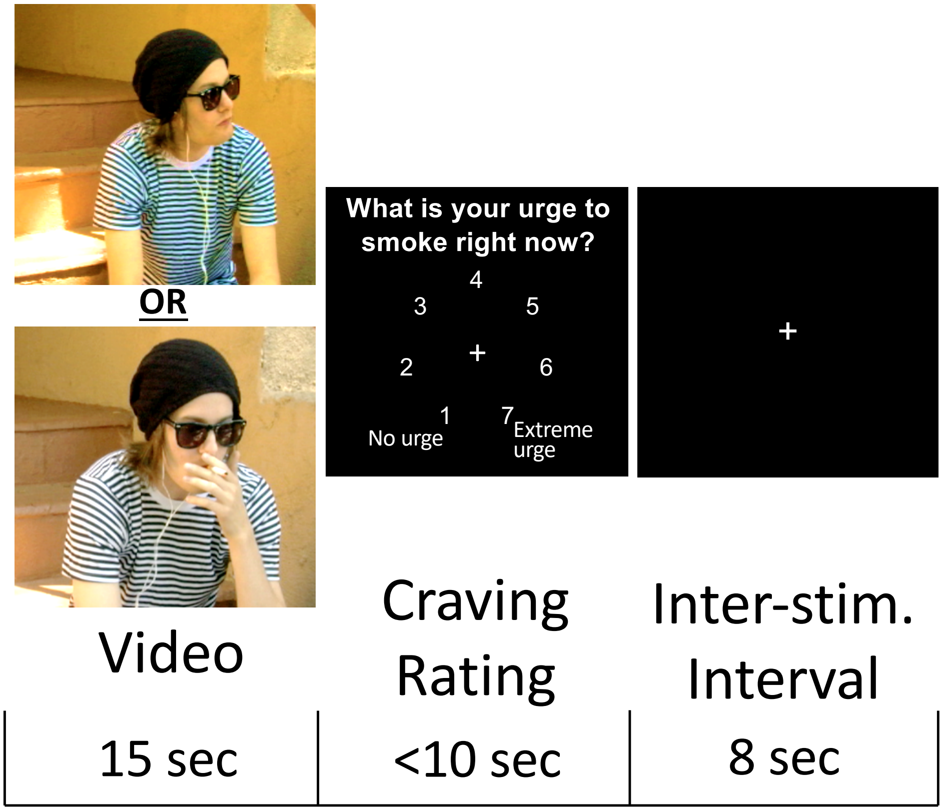

Supplementary Figure 2. Schematic of cue-induced craving task used during fMRI scanning. On each trial, participants viewed a 15 second video clip of either someone smoking a cigarette (smoking cue) or someone in the same context who is not (neutral cue). Subsequently, they provided a rating as to how much they felt the urge to smoke in that moment using a trackball (1 through 7 rating, with 1=”No urge” and 7=”Extreme urge”). When the rating screen appeared, the trackball cursor was positioned on crosshairs in the center of a circle, equidistant to each of the seven rating options to minimize bias towards any particular response. Upon making their response by clicking on a number, the number turned yellow for 1 second prior to advancing to an 8 sec inter-stimulus interval (ISI) during which crosshairs appeared in the center of the screen. If participants did not respond within ten seconds, the program would automatically advance to the ISI. Shown are individual frames from video clips used in the study, which were produced by a professional video production company. Individuals in the videos were professional actors who provided consent for publication of their images.

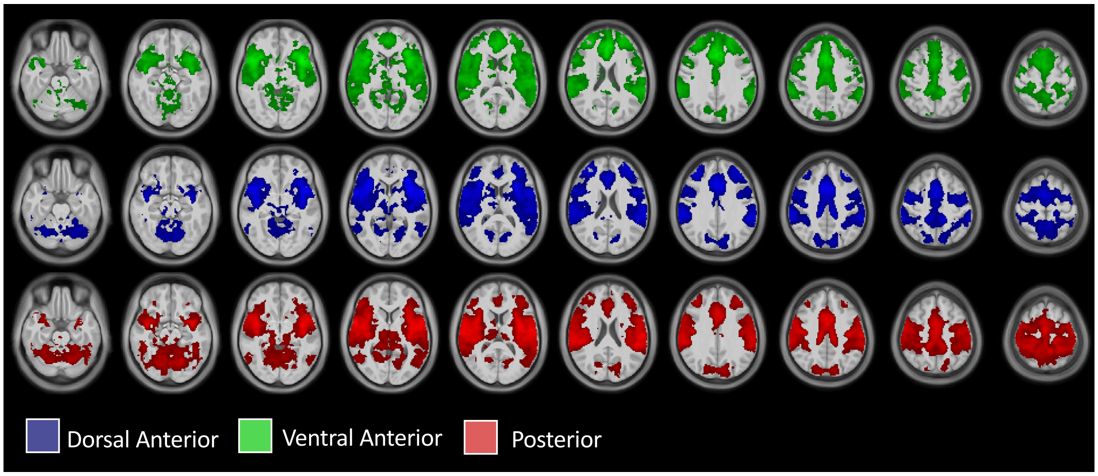

Supplementary Figure 3. Resting state functional connectivity of insula subregions. Group connectivity maps for each left insula seed (dorsal anterior in blue, ventral anterior in green and posterior in red) are overlaid on the group mean anatomical T1 image across the three rows (height threshold: Z>3.1, corrected for cluster size, p<0.05). Images shown in radiological orientation (left is right).

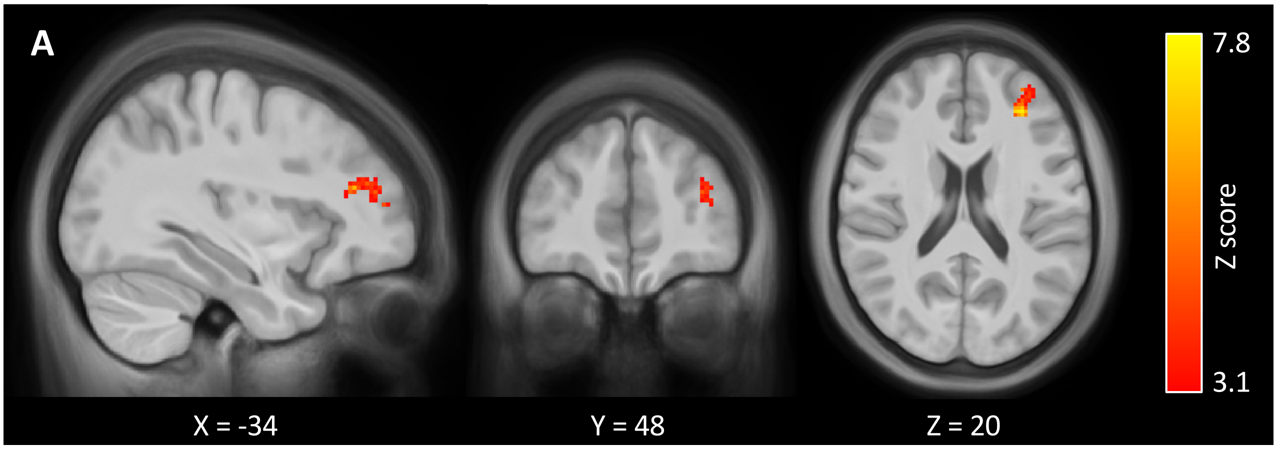

Supplementary Figure 4. Relationship between nicotine dependence (FTND total score) with cue-induced fMRI activation. A. A whole-brain, voxel-wise cluster-corrected analysis indicated a positive relationship between FTND and the contrast of smoking vs. neutral cues in the left middle frontal gyrus. Figure shows thresholded statistical maps (p<0.001, cluster corrected) displayed on the average of spatially normalized T1-weighted images (to MNI template) across participants (N=48). Brain images are presented in radiological convention (right=left).

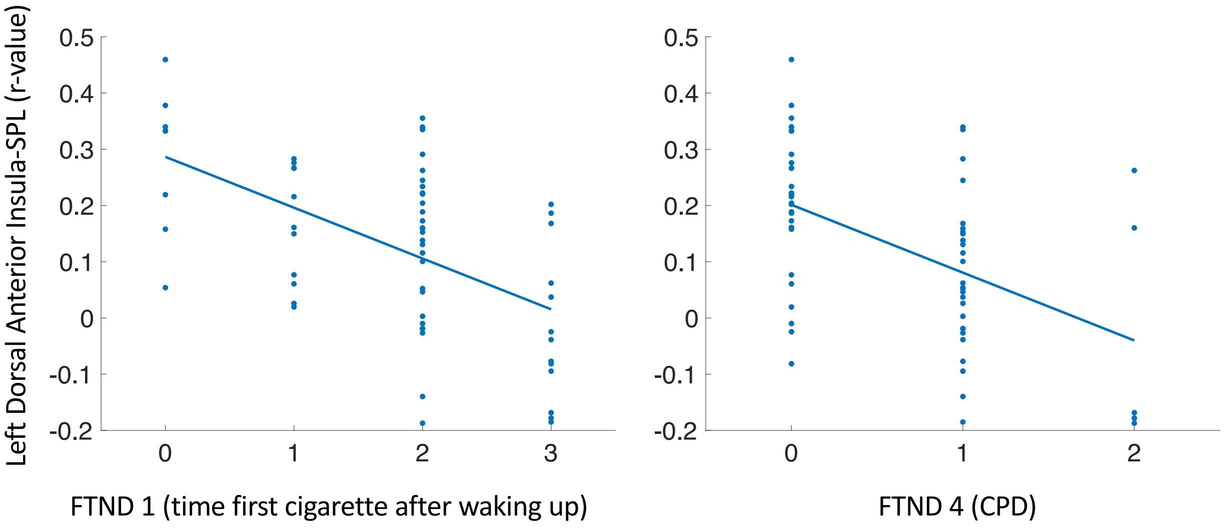

Supplementary Figure 5. Scatterplots indicating negative relationships between left dorsal anterior insula-SPL functional connectivity and two items from the Fagerstrom Test of Nicotine Dependence (FTND). The first item in the FTND scale (FTND1) assesses the time until the first cigarette of the day after waking (plotted increments: 0 = within 5 minutes, 1 = 6-30 minutes, 2 = 31-60 minutes, 3 = after 60 minutes). The fourth FTND item (FTND4) asks about the number of cigarettes smoked per day (CPD) (plotted increments: 0 = 10 or less, 1 = 11-20, 2 = 21-30 cigarettes). Statistics are provided in Supplementary Table 1 below)

Supplementary Table 1.

ANOVA omnibus tests: Resting state functional connectivity of insular subregions and SPL with individual items on FTND scale

	**L Dorsal Anterior**		**L Ventral Anterior**		**R Dorsal Anterior**		**R Ventral Anterior**	
	**p**	**η²**	**p**	**η²**	**p**	**η²**	**p**	**η²**
**FTND1**	***0.017***	0.061	***0.011***	0.067	***0.032***	0.049	***0.020***	0.068
**FTND2**	0.129	0.024	0.408	0.007	0.432	0.006	0.980	0.000
**FTND3**	***0.020***	0.058	0.779	0.001	***0.048***	0.041	0.438	0.007
**FTND4**	***0.005***	0.085	***0.009***	0.070	***0.015***	0.064	0.132	0.028
**FTND5**	0.888	0.000	0.175	0.018	0.282	0.012	0.267	0.015
**FTND6**	0.557	0.003	0.644	0.002	0.624	0.002	0.770	0.001

Controlling for Age and mFD

p < .05

ANOVA Omnibus tests: Cue-induced activation (smoking vs. neutral cues) of insular subregions with individual items on FTND scale

	**L Dorsal Anterior**		**L Ventral Anterior**		**R Dorsal Anterior**		**R Ventral Anterior**	
	**p**	**η²**	**p**	**η²**	**p**	**η²**	**p**	**η²**
**FTND1**	0.326	0.015	0.374	0.013	0.447	0.012	0.777	0.002
**FTND2**	0.352	0.014	0.295	0.017	0.930	0.000	0.842	0.001
**FTND3**	0.077	0.051	0.105	0.043	0.667	0.004	0.302	0.022
**FTND4**	0.093	0.045	***0.022***	0.088	0.695	0.003	0.321	0.021
**FTND5**	***0.001***	0.217	***0.001***	0.214	***0.028***	0.108	***0.021***	0.118
**FTND6**	0.092	0.046	0.106	0.043	0.823	0.001	0.565	0.007

Controlling for Age and mFD

p < .05

FTND1 - How soon after you wake up do you smoke your first cigarette?
FTND2 - Do you find it difficult to refrain from smoking in places where it is forbidden?
FTND3 - Which cigarette would you hate most to give up? (a. The first one in the morning, b. Any other)
FTND4 - How many cigarettes per day do you smoke?
FTND5 - Do you smoke more frequently during the first hours after waking than during the rest of the day?
FTND6 - Do you smoke if you are so ill that you are in bed most of the day?

Supplementary Table 2.

Relationship between cue-induced activation (smoking vs. neutral cues) in anterior insular subregions and resting state functional connectivity of these regions and SPL. Shown are results from separate analyses for each *a priori*-defined anterior insula subregion.

Insula Subregion	SS	F	p	Effect size η^2p
L Dorsal Anterior-SPL	3776	6.75	0.013[a]	0.133
R Dorsal Anterior-SPL	1021	3.03	0.089	0.064
L Ventral Anterior-SPL	977	1.68	0.202	0.037
R Ventral Anterior-SPL	108	0.26	0.612	0.006

Note. Statistical values for each subregion are from separate linear models that included age and mean FD as covariates. SS – sums of squares. General Linear Model details in methods.
[a]Statistically significant at $p<0.05$

Supplementary Table 3.

Post-hoc examination of effects of sex and cannabis use on cue-induced activation (smoking vs. neutral cues) and resting state functional connectivity. No statistically significant relationships were found.

ANOVA Omnibus tests	Cue-induced activation	RSFC
	p value	p value
R Dorsal Anterior		
Sex	0.14	0.82
Cannabis Use (days in the last 30)	0.80	0.74
Cannabis Use (grams/week)	0.62	0.27
L Dorsal Anterior		
Sex	0.14	0.43
Cannabis Use (days in the last 30)	0.66	0.38
Cannabis Use (grams/week)	0.72	0.78
L Ventral Anterior		
Sex	0.14	0.26
Cannabis Use (days in the last 30)	0.60	0.53
Cannabis Use (grams/week)	0.76	0.55
R Ventral Anterior		
Sex	0.14	0.83
Cannabis Use (days in the last 30)	0.66	0.20
Cannabis Use (grams/week)	0.46	0.54

Note. Statistical values for each subregion are from separate linear models that included FTND score, age and mean FD as covariates in addition to sex and cannabis use. SS – sums of squares. RSFC – resting state functional connectivity. General Linear Model details in methods.

[a]Statistically significant at $p<0.05$
